# Trajectory-driven computational analysis for element characterization in *Trypanosoma cruzi* video microscopy

**DOI:** 10.1371/journal.pone.0304716

**Published:** 2024-06-03

**Authors:** Geovani L. Martins, Daniel S. Ferreira, Claudia M. Carneiro, Nivia C. Nogueira-Paiva, Andrea G. C. Bianchi

**Affiliations:** 1 Postgraduate Program in Computer Science, Federal University of Ouro Preto, Ouro Preto, MG, Brazil; 2 Department of Computing, Federal University of Ouro Preto, Ouro Preto, MG, Brazil; 3 Department of Computing, Federal Institute of Education, Science, and Technology of Ceará, Maracanaú, CE, Brazil; 4 Nucleus of Biological Sciences Research, Federal University of Ouro Preto, Ouro Preto, MG, Brazil; 5 Department of Clinical Analysis, Federal University of Ouro Preto, Ouro Preto, MG, Brazil; Oswaldo Cruz Foundation, BRAZIL

## Abstract

Optical microscopy videos enable experts to analyze the motion of several biological elements. Particularly in blood samples infected with *Trypanosoma cruzi* (*T. cruzi*), microscopy videos reveal a dynamic scenario where the parasites’ motions are conspicuous. While parasites have self-motion, cells are inert and may assume some displacement under dynamic events, such as fluids and microscope focus adjustments. This paper analyzes the trajectory of *T. cruzi* and blood cells to discriminate between these elements by identifying the following motion patterns: collateral, fluctuating, and pan–tilt–zoom (PTZ). We consider two approaches: i) classification experiments for discrimination between parasites and cells; and ii) clustering experiments to identify the cell motion. We propose the trajectory step dispersion (TSD) descriptor based on standard deviation to characterize these elements, outperforming state-of-the-art descriptors. Our results confirm motion is valuable in discriminating *T. cruzi* of the cells. Since the parasites perform the collateral motion, their trajectory steps tend to randomness. The cells may assume fluctuating motion following a homogeneous and directional path or PTZ motion with trajectory steps in a restricted area. Thus, our findings may contribute to developing new computational tools focused on trajectory analysis, which can advance the study and medical diagnosis of Chagas disease.

## Introduction

The motility of the parasite *Trypanosoma cruzi* (*T. cruzi*), which is the etiological agent of Chagas disease [1], is an important visual stimulus in optical microscopy analyses. The low contrast presented by this microorganism makes its motion an essential indicator of its presence in the analysis of blood samples without staining techniques. However, *T. cruzi* is not the only dynamic element observed during these analyses. Blood cells also show some dynamic responses to different stimuli. Except in situations in which the parasitologist is fatigued or inexperienced, which often leads to incorrect analyses and diagnoses, the dynamic behavior of these elements differentiates them.

Trypanosomatids are a group of highly versatile microswimmer parasites. The motility of these parasites depends greatly on their morphology (size, shape, propulsion mechanism) and on the environment in which they move. Some works present important insights into the motility of trypanosome species, such as *T. brucei* [2–7], *T. carassii* [8], *T. congolense*, *T. evansi*, and *T. vivax* [9]. *T. cruzi* motility studies focus on the epimastigote [10–12] and trypomastigote [13] stages of its life cycle. Video microscopy and the vectorial analysis of *T. cruzi* trajectories indicate the alternation of rectilinear and intricate motility paths. In addition, this parasite’s flagellar and ciliary beating generates different distances traveled [10]. The speed during its tumbling motion is less than that of the nearly rectilinear persistent motion [11]. From the perspective of diffusive motion patterns, the spreading of its trajectory is strongly superdiffusive for short-times, and the speed time series present long-range correlations [12]. Various factors can affect *T. cruzi* motility [14, 15]. A recent finding establishes that *T. cruzi* trypomastigotes can detect the presence of mammalian cells and change their motility patterns [13].

The parasitemia level is high during the acute phase of Chagas disease, favoring the detection of motile trypomastigotes by blood microscopy. In contrast, the diagnosis in the chronic phase relies on serological methods [16]. When laboratory procedures require examining samples containing live parasites, they typically consider motion in their analysis, such as detecting motile trypomastigotes in newborns from mothers with Chagas disease [17]. Although the motion is an intrinsic and indispensable biological aspect of *T. cruzi*, this characteristic still needs to be better explored and investigated using computational approaches to aid the study and medical diagnosis of Chagas disease. Among the approaches used to detect these parasites [18–31], few studies consider motion [18, 19, 25, 26, 29, 31]. The dynamic context involved in the optical microscopy of blood samples infected with *T. cruzi* parasites is one of the challenges encountered by these approaches, which can be sensitive to different stimuli [29]. To the best of our knowledge, the motion-based approaches focus on detecting parasites without particularly identifying blood cells. However, cells are elements that can introduce false positives. Tracking these elements and analyzing their trajectories can improve detection performance.

**Fig 1:**
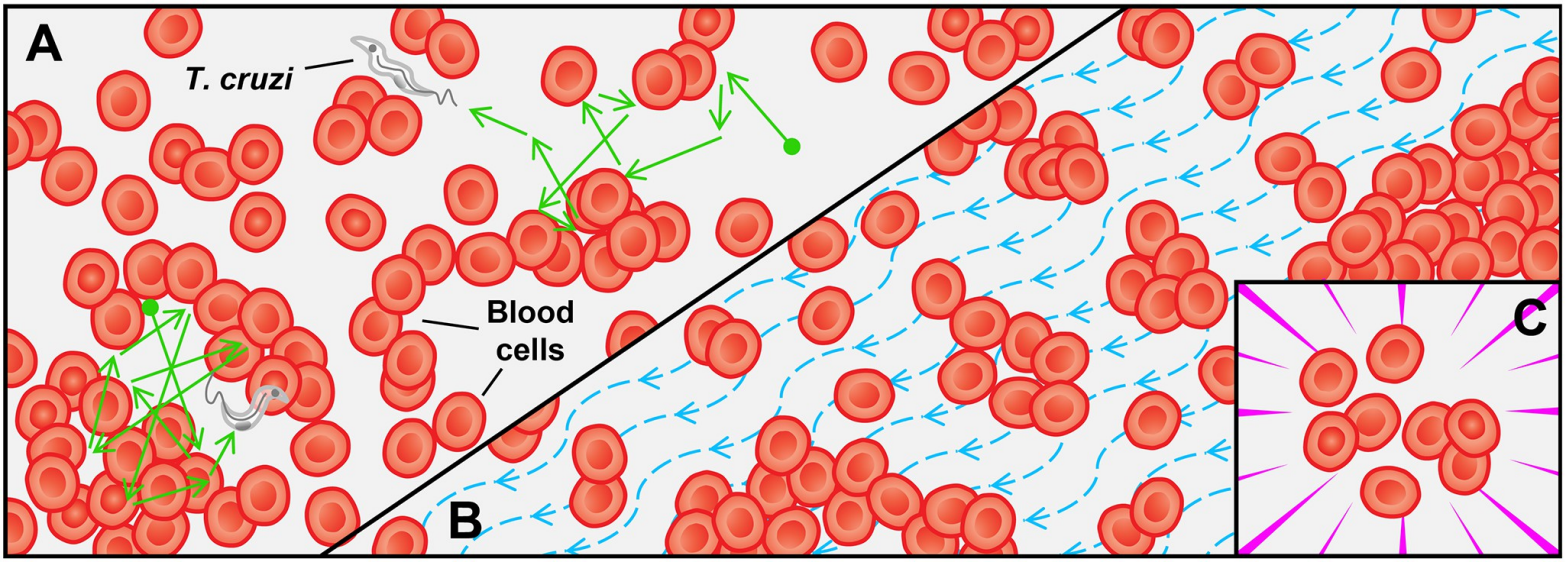
Dynamism in video microscopy of *T. cruzi* parasites. Three motions are illustrated: (A) collateral motion observed during the locomotion of *T. cruzi* parasites between cells; (B) the fluctuating motion of cells or other artifacts present in the blood; and (C) PTZ motion due to microscope or camera focus adjustments.

Fig 1 shows the movement patterns observed in *T. cruzi* parasitological analysis videos. Collateral motion corresponds to stimuli perceived during the locomotion of *T. cruzi*, which interacts with neighboring cells and fluids. It is not the parasite’s intrinsic motion but a motion resulting from collisions with other elements in the scene. Fluctuating motion is mostly identified in cells suspended in the blood sample. Although cells do not have a self-motion, they are susceptible to dynamic events that make them vulnerable to sudden movements. An example is the handling of moving parts of the microscope, which can destabilize the cells. Pan–tilt–zoom (PTZ) motion is related to the focus adjustments of the microscope lenses or even the camera that captures the visual field. In this case, static elements can express apparent motion due to the focus adjustments. Generally, this type of motion influences the performance of computational approaches that extract the spatio-temporal features of the scene.

The study of the trajectory of the elements involved in the parasitological analysis of *T. cruzi* can reveal interesting insights about the reported motion patterns and thus contribute to developing new applications. While parasites are mobile elements by nature, cells are inert and start to move under the influence of dynamic events, as observed in optical microscopy videos. We hypothesize that the dynamic context emphasized by Martins et al. [29] generates distinct trajectory patterns between the biological elements and may be explored to characterize *T. cruzi* and blood cells by computational approaches. While collateral motion-stimulated blood cells exist, our study is restricted to cells that do not interact with parasites. This enables us to identify the regular dynamic patterns of cells. By investigating how the trajectories differ, our contributions are the following: i) an approach to distinguishing the element that performs the motion based on its trajectory; ii) an approach to analyzing the type of cell motion; and iii) a descriptor for measuring the standard deviation of steps in different trajectory segments at constant time intervals. The following sections present the proposed methodology used to test our hypothesis, the obtained results, a discussion, and conclusions.

## Materials and methods

**Fig 2:**
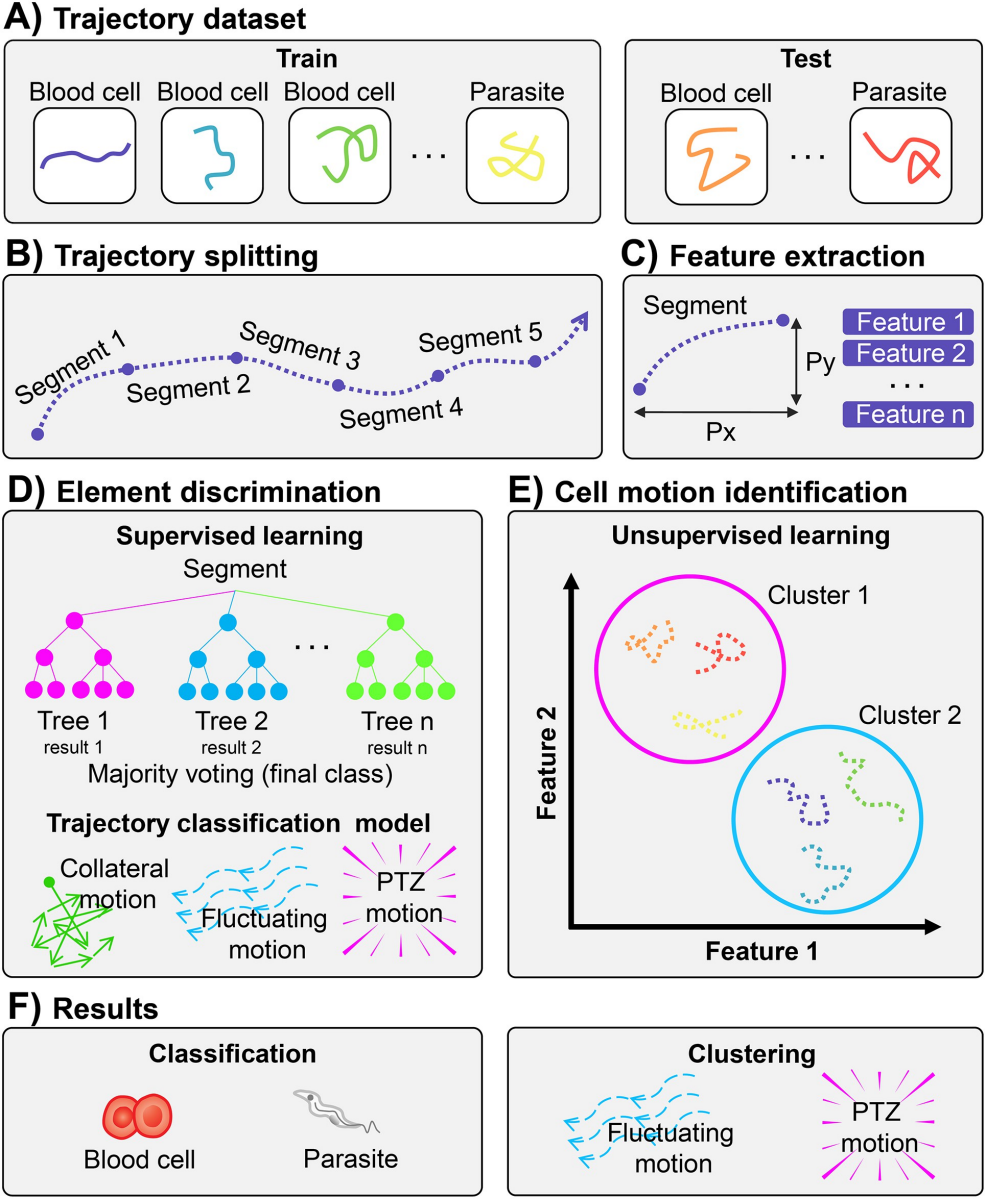
Overview of trajectory analysis. (A) The trajectories of parasites and cells are split to form training and test datasets. (B) The trajectories are partitioned into segments for a better analysis of their behavior. (C) Features are extracted from trajectory segments. (D) Approach to discriminate between elements. Supervised learning classification models learn the training dataset’s motion patterns and categorize the test dataset’s trajectory segments. (E) Approach to identify cell motion patterns. Unsupervised learning models identify hidden patterns in training and test trajectory segments and group similar segments into a cluster. (F) We evaluated the performance of the experiments according to the classification accuracy (distinguishing between parasites and cells) and the clustering quality (distinguishing between fluctuating and PTZ motion).

We present the pipeline of this work in Fig 2. We propose two experimental approaches. The first one defines a supervised tree-based method to classify cells and parasites based on motion parameters. The second one defines a clustering method to differentiate the two cell motions (fluctuating/PTZ).

### Dataset

Here, we utilized the dataset introduced by Martins et al. [29], which comprises optical microscopy videos of blood samples and trajectories of *T. cruzi* trypomastigotes. Videos are up to 10 seconds long, with a frame rate of approximately 30 frames per second (fps) and a resolution of 640 x 480 pixels. The training and testing videos have distinct parasite trajectories. We performed the same procedure as Martins et al. [29] to generate the trajectories of the cells. We randomly selected three cells for each parasite in the video as our sample. It is important to emphasize that no chosen cell interacts with the parasites along its trajectory. Then, we labeled these cells using the Tracker tool [32], which is free and open source, and an expert validated the trajectories obtained. A trajectory is a sequence of positions of an element that moves within a spatio-temporal context [33, 34]; it can be represented as *traj* = < *p*_1_, *p*_2_, *p*_3_, …, *p*_*n*_ > where *p*_*n*_ = (*x*_*n*_, *y*_*n*_, *t*_*n*_) is the *n*th trajectory point, that is, the point in frame *n*. The (*x*_*n*_, *y*_*n*_) coordinates represent the spatial location of *p*_*n*_; *x* and *y* represent the horizontal and vertical coordinates, respectively, in video pixels. *t*_*n*_ is the time at which *p*_*n*_ is regularly recorded. Since the videos were taken at 30 frames per second, $\Delta t=0.0\bar{3}\;s$ for two consecutive frames between 1 and *n*.

### Trajectory splitting

One of the techniques used in the pre-processing of trajectory data is splitting, which makes it possible to prepare these data for future classification and clustering tasks. Trajectory splitting reduces the computational complexity and allows better analysis and discovery of trajectory patterns [33]. We consider time intervals of the same duration for trajectory splitting. Thus, a segment is part of a complete trajectory, and it is represented as *traj*_*seg*_ = < *p*_*i*_, *p*_*i*+1_, *p*_*i*+2_, …, *p*_*i*+(*k*−1)_ > where *p*_*i*_ is the initial point of the trajectory segment, and *k* is the total number of successive points to be considered.

### Feature extraction

We propose the trajectory step dispersion (TSD) descriptor to estimate the step length dispersion of trajectory segments. Since *T. cruzi*’s motion is conspicuous in the scene [29] and this microorganism is the only one with self-motion, we consider that parasites and cells can be distinguished by analyzing the variance between different segments of their trajectories at constant time intervals. The TSD can be computed as follows for a trajectory segment consisting of *k* points:

$$TSD=\sqrt{\frac{1}{k-1}\sum_{i=1}^{k-1}{\left(l_{i}-\bar{l}\right)}^{2}},$$ (1)

where the step length is *l*_*i*_ = *p*_*i*+1_ − *p*_*i*_ ∀*i* ∈ [1, *k* − 1], and the mean is $\bar{l}=\frac{1}{k-1}\sum_{i=1}^{k-1}l_{i}$.

Since the mean speed and mean square displacement power-law exponent (λ-MSD) are relevant to describing the motility patterns of *T. cruzi* trypomastigotes [13], we consider both in our study.

### Element discrimination

Our classification task is a binary classification problem in which we need to label a trajectory segment according to whether it was followed by a parasite or a blood cell. We used three variations of tree-based classifiers: the decision tree [35, 36], which is a simple decision making-diagram; random forest [37, 38], which consists of a collection of decision trees combined using averages or majority rules at the end of the decision-making process; and extreme gradient boosting (XGBoost) [39], which implements the concept of gradient boosting using more precise approximations to find the best tree model.

### Cell motion identification

We combine cell trajectory segments from the training and test datasets. As blood cells can perform two motions, we propose trajectory clustering to group similar trajectory segments and thus investigate the mobile behavior of each cluster. The best performance occurs with the maximization of the homogeneity within and heterogeneity between clusters [40]. We use the spectral clustering method [41, 42], since this method outperformed the classical algorithms on a wide variety of datasets [43]. Spectral clustering is based on a graph partitioning problem in which nodes in the graph are associated with minimum distances. It requires the number of clusters to be specified a priori; this number corresponds to the cell motion patterns to be investigated.

### Statistical analysis

We evaluate the relationship between features through correlation analysis. We compute the pairwise correlation of features using the Pearson method [44]. The value of Pearson’s correlation coefficient varies from −1 to + 1, where −1 indicates a strong negative correlation, and + 1 indicates a strong positive correlation. If 0, there is no relationship between the features. We also analyze the probability distribution of parasite and blood cell trajectory features based on kernel density estimation (KDE). We estimate density using Gaussian kernels and Scott’s rule to get the bandwidth [45]. From the perspective of diffusion models, we explore the behavior of MSD curves, as the increase in this statistical measure with time can be fitted to the power law *MSD*(*t*) ∝ *t*^λ^ (λ-MSD). The value of λ defines whether the motion is subdiffusive (λ < 1), diffusive (λ = 1), or superdiffusive (λ > 1) [46].

### Metrics

Accuracy, precision, recall, and *F*_1_ metrics evaluate the classification performance [47]. The clustering experiments are evaluated in terms of the silhouette coefficient [48, 49].

## Results and discussion

We analyze trajectory segments of 1 second, which correspond to sequences of 30 trajectory points (*k* = 30). We define this *k* value based on the video frame rate of 30 fps. Martins et al. [29] introduced sequences of 25 frames to detect *T. cruzi* motion. Arias-del-Angel et al. [13] used a 0-2s interval to define parasite motility patterns. Thus, our parameter *k* is within an acceptable range in the literature. We also consider that smaller or larger sequences may have a loss in the motion definition.

**Fig 3:**
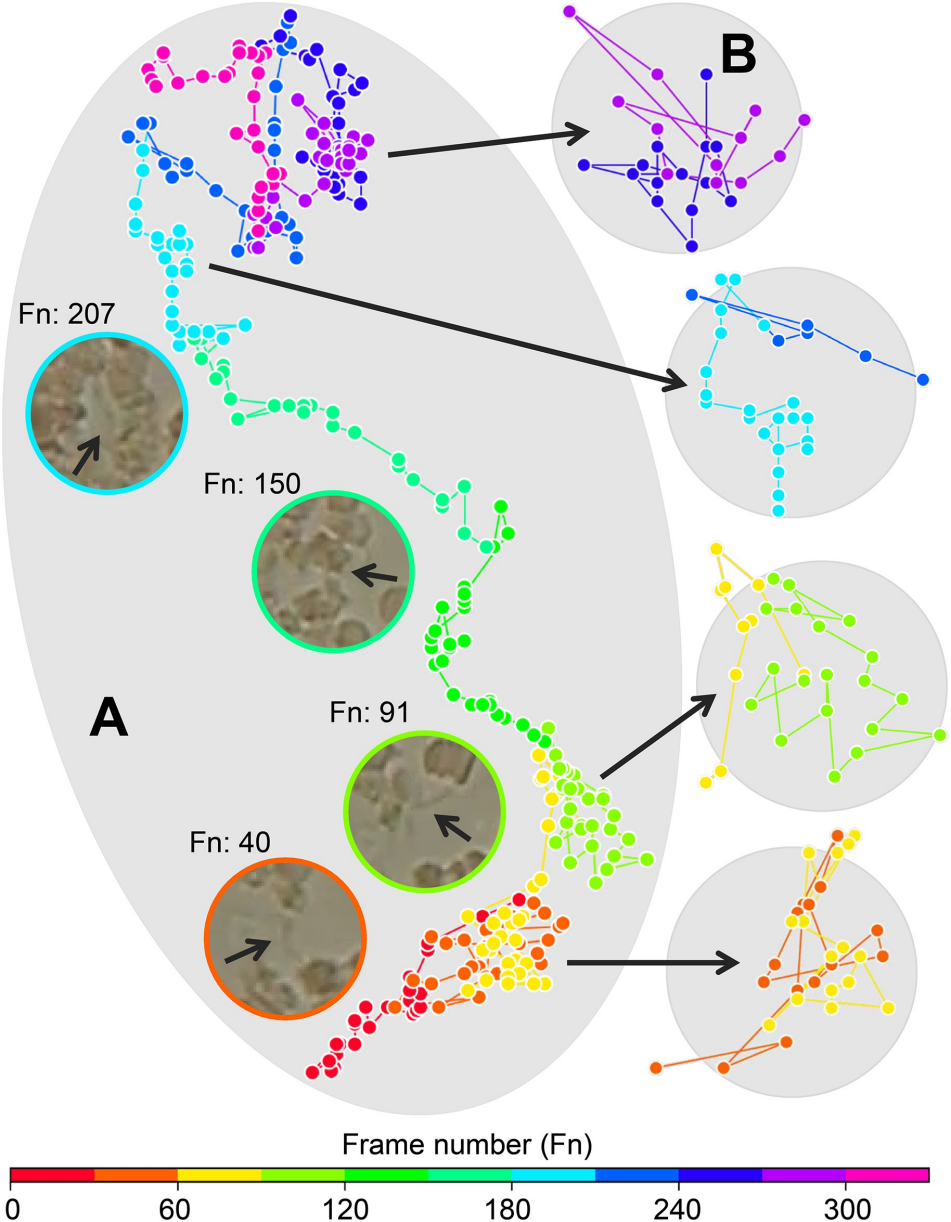
Trajectory segments of the *T. cruzi* parasite obtained from the sequential and random splitting strategies. (A) Sequential split: segments are generated sequentially along the complete trajectory at regular intervals of *k* points. Different colors in the complete trajectory represent the segments. The parasite region is highlighted for some trajectory segments. (B) Random split: segments are obtained by randomly choosing a starting point on the trajectory and the *k* − 1 following points.

We present the complete trajectory of a *T. cruzi* parasite and the trajectory splitting strategies in Fig 3. The sequential splitting approach generated a total of 548 trajectory segments, including 432 (blood cells—324 and parasites—108) training and 116 (blood cells—87 and parasites—29) test segments. We applied random splitting to the training segments only as a data augmentation strategy to reduce overfitting in the element discrimination experiment. This procedure can increase the decision capacity of the trained models [50], as shown in the next section. Fig 3A also shows the surrounding region of the parasite in some video frames. It is important to note that the *T. cruzi*’s low contrast makes it difficult to locate. Its shape is not consistent; it varies along the trajectory. This is especially true when it overlaps with cells, as in frame 150. In addition, the surrounding elements change with the displacement of the parasite, which affects its trajectory. The parasite starts its path by colliding with groups of cells located above and below, as shown in frames 40 and 91. The flagellar beats are intense, and the shocks propel the parasite against the opposite group of cells. Then the parasite swims persistently, crossing restricted spaces between cells. We observed a gradual increase in the number of blood cells around the parasite, including a confinement scenario in frame 207. The last segments depict the parasite’s interactions in another containment situation between cells. The trajectory is limited to a certain region with circular aspect.

**Fig 4:**
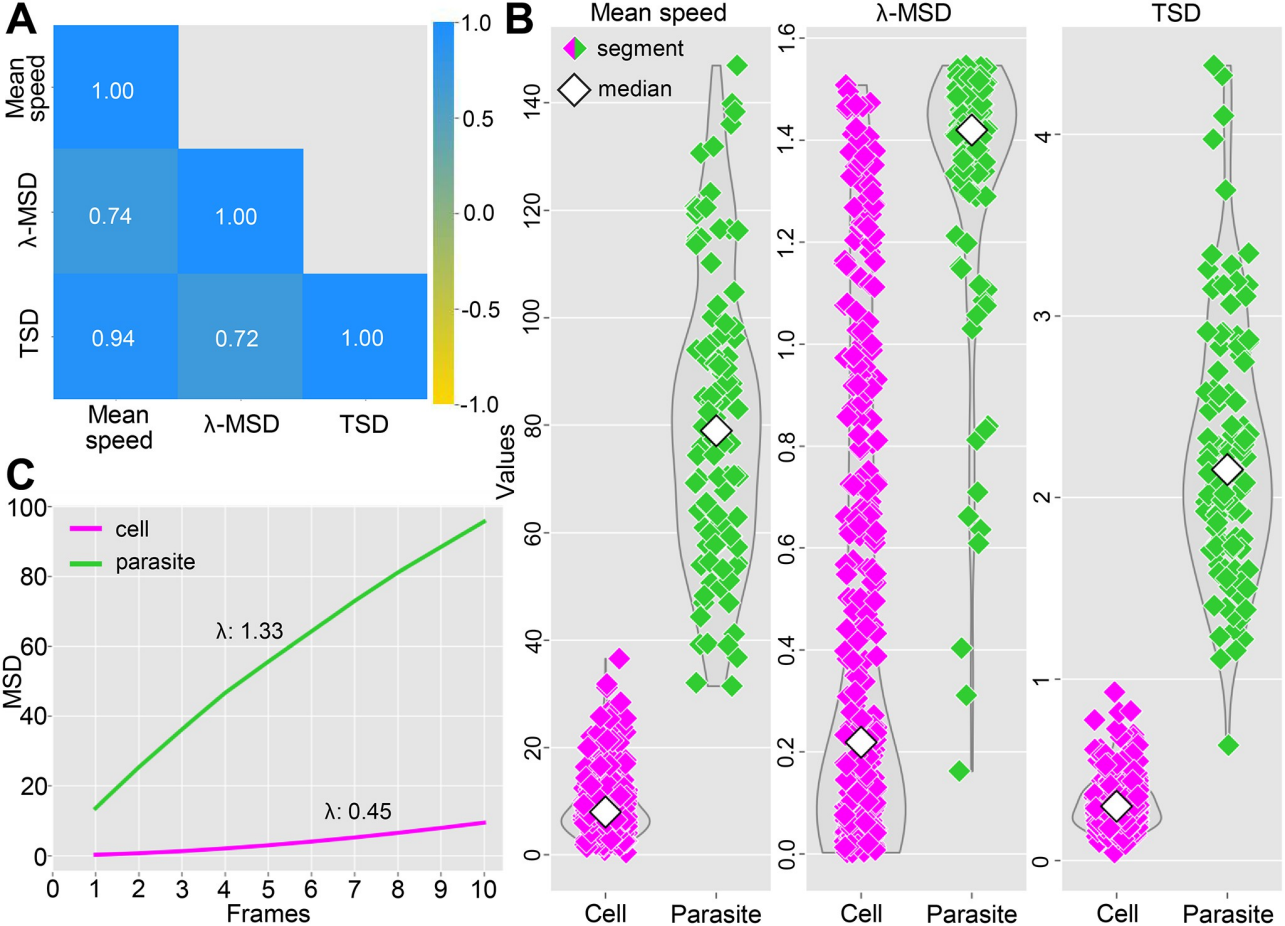
Feature analysis from training trajectory segments. (A) Pairwise correlation of features. The values correspond to the correlation coefficient: -1.00 indicates a perfect negative correlation, +1.00 indicates a perfect positive correlation, and 0 indicates no correlation between the features. (B) Distribution and probability density of trajectory segments by feature. Blood cell and parasite segments are represented by pink and green rhombuses, respectively. White rhombuses indicate the median of the distribution. The width of each curve corresponds to the approximate frequency of trajectory segments in the region. (C) Mean squared displacements (MSDs) versus the number of video frames. The curves summarize the computed MSD at intervals of 1 to 10 video frames (∀*j* ∈ [1, 10] in *MSD*(*t*_*j*_)) for all trajectory segments.

We analyze the features extracted from the training trajectory segments in Fig 4. Fig 4A shows the pairwise correlation of features using the Pearson method [44]. All feature pairs are positively correlated, with values above 0.70. The mean speed is highly correlated with the TSD, while the λ-MSD has a moderate correlation with these two features. Fig 4B presents the data distribution by feature. The trajectory segments of parasites are more dispersed compared to the cells. The disordered flagellar beating and intense collisions with cells contribute to the distribution of trajectory segments in high-value ranges. The cells have trajectory segments concentrated in low-value ranges for the mean speed and TSD, which indicates that the trajectory steps are restricted and tend to preserve the overall behavior of the trajectory. Although the two elements have similar dispersions of the λ-MSD, the medians of the distributions occupy opposite regions in the graph. Fig 4C confirms the superdiffusion behavior of the parasites since the trajectory segments had a λ-MSD that was greater than 1. On the other hand, the λ-MSD of the cells of less than 1 indicates that they perform subdiffusive motion [46]. This result was expected since the cells do not have a self-motion like the parasites. Note that although cells tend to subdiffusive motion, they can occasionally have λ-MSD greater than 1. Parasites in confinement may have difficulty in locomotion, exhibiting subdiffusive behavior.

### Element discrimination

Our classification experiment aims to study the problem of element discrimination based on trajectory analysis. Initially, the hyperparameters of the classification algorithms were tuned using grid search with 4-fold cross-validation on the training dataset. We repeated this experiment 10 times, training the classification algorithms with different arrangements of trajectory segments generated with random splitting. This splitting strategy increased the amount of training data and provided balanced classes, reducing overfitting during model building. We established 30 segments for each parasite trajectory and 10 for each cell trajectory per video. Thus, the classifiers were trained with 780 trajectory segments (blood cells—390 and parasites—390) in each repetition. All the features extracted from the trajectory segments have been normalized. The mean accuracy values for cross-validation were above 0.980 for the three classifiers used in our experiment. This result shows that the models generated with the adjusted parameters have valuable generalization capacities for new trajectory segments.

Once the model has been trained, the resulting model can predict the labels of the test trajectory segments. These segments were generated with sequential splitting. Thus, we obtained 116 trajectory segments (blood cells—87 and parasites—29). The accuracy of each classifier is shown in Table 1. Note that we perform predictions with isolated features and with all of the features (the feature collection). The feature collection and TSD presented the best results, achieving the best performance with the XGBoost classifier. The feature collection also obtained an accuracy of 0.966 with the decision tree classifier. The λ-MSD presented the worst performance out of all the features, obtaining the lowest accuracy of 0.440 with the decision tree classifier. Considering that the XGBoost algorithm obtained the highest accuracy in two analyzed cases, we present the results for the precision, recall, and *F*_1_ metrics in Table 2. The feature collection and TSD presented the same performance for all metrics. Maximum precision means that the model is always correct when it predicts that the trajectory segment is from a parasite. The low precision for the λ-MSD may explain the low accuracy observed in Table 1. We analyzed the relative importance of each feature in the feature collection for model prediction and found that TSD contributes 99.8% of the final result. The TSD descriptor was also the most relevant feature for the other classifiers, indicating that TSD is a valuable descriptor that can be used to identify *T. cruzi* parasites in video microscopy.

**Table 1:** Accuracy results for test trajectory segments for different tree-based classifiers.

	Feature collection	Mean speed	λ-MSD	TSD
Decision tree	**0.966**	0.888	0.440	0.948
Random forest	0.957	0.862	0.543	0.931
XGBoost	**0.966**	0.888	0.509	**0.966**

**Table 2:** XGBoost classifier performance metrics.

	Precision	Recall	*F*_1_
Feature collection	**1.000**	**0.862**	**0.926**
Mean speed	0.767	0.793	0.780
λ-MSD	0.274	0.586	0.374
TSD	**1.000**	**0.862**	**0.926**

**Fig 5:**
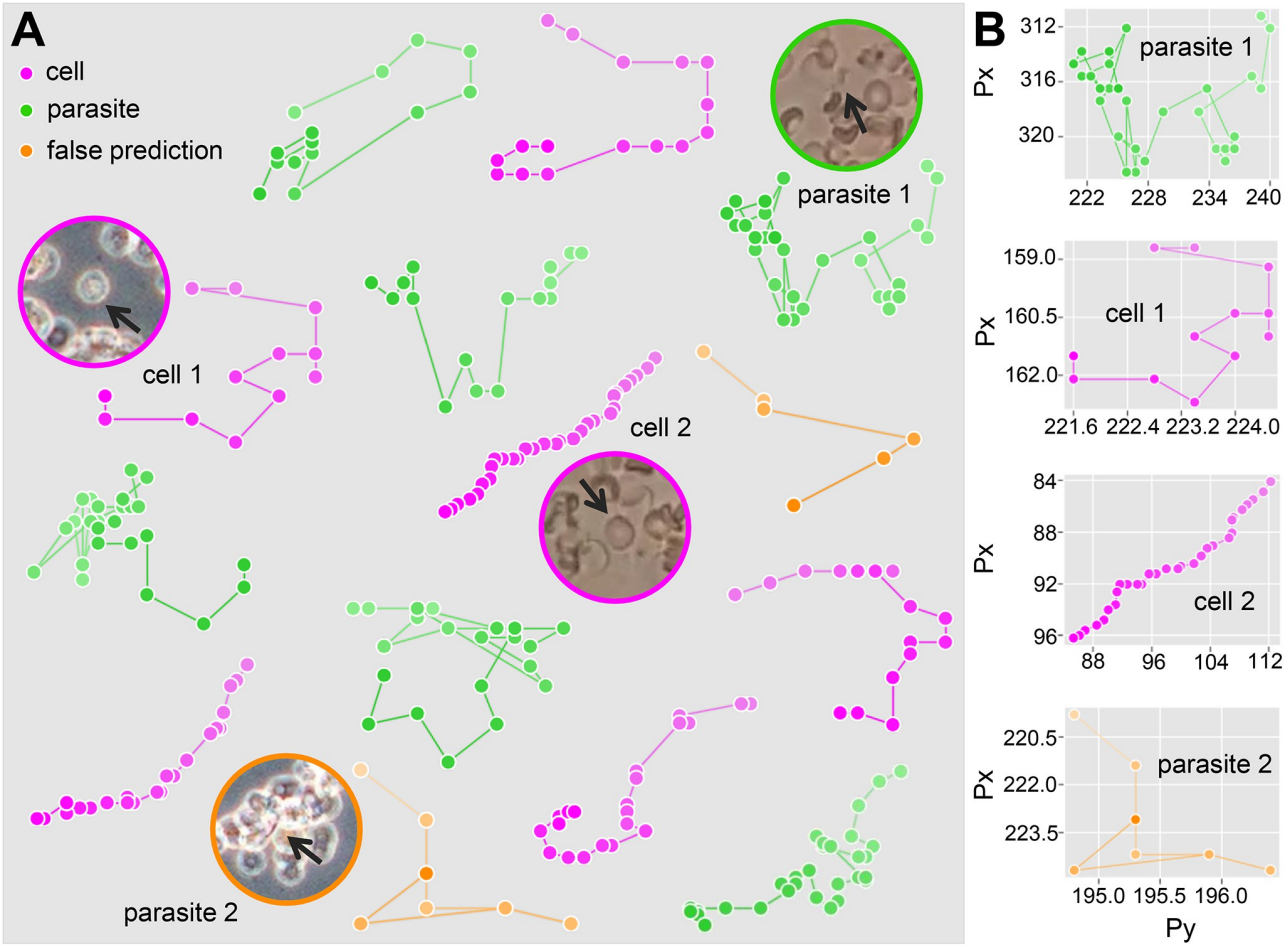
Qualitative analysis of the XGBoost classifier results in the feature collection experiment. (A) Trajectory segments by predicted class. The lighter tones of each segment indicate the beginning of the trajectory, and the darker tones indicate the end. The moving element region is highlighted for some trajectory segments. The arrow indicates the precise location of the element. (B) Comparison of the distance traveled by the elements.

The qualitative analysis of the results obtained with the XGBoost classifier in the feature collection experiment shows distinct patterns for parasites and blood cells. Fig 5A highlights some trajectory segments and the predicted class. The trajectory segments of the *T. cruzi* parasite are characterized by high dispersion and irregularity in the displacement. The pattern demonstrates the parasite’s intrinsic swimming motion, which already has a certain randomness, and the influence of its interactions with the environment; for example, parasite 1 is surrounded by cells. These aspects highlight the collateral motion of the parasites in the microscope’s visual field. There are also cell trajectory segments with more dispersed patterns, such as cell 1 in Fig 5A. However, Fig 5B shows that the displacement of these cells is smaller than the parasites. Cell 1 presents some trajectory steps with a perpendicular shape, in addition to overlapping points, indicating the absence of motion. On the other hand, cell 2 maintains a uniform and directional motion pattern, covering a greater distance than cell 1. Parasite 2 is in a confinement and cell overlap scenario, making locomotion difficult. The trajectory segment of parasite 2 has many overlapping trajectory points, similar to cell 1, causing the false prediction.

### Cell motion identification

We analyzed blood cell motion patterns using clustering experiments. We generate a single dataset with all the cell trajectory segments of the training and test datasets. The segments were obtained using the sequential splitting strategy, which provided 411 trajectory segments. The experiments were performed with the spectral clustering algorithm, with the number of clusters equal to two. The clustering performance is given in terms of the silhouette coefficient for the feature collection and feature pairs in Table 3. We found silhouette coefficients of around 0.67, with no values close to zero or negative. These results indicate that the generated clusters have reasonable cohesion and separation [51]. The TSD + λ-MSD feature pair presented the best silhouette coefficient compared to the other feature combinations. We also investigated the percentage of trajectories with variation in motion pattern, which we called bi-labeled trajectories. Considering that a moving cell tends to preserve its motion along the trajectory, a percentage equal to or close to zero indicates a better clustering. Trajectories without variation in the segment labeling reveal more consistent dynamic patterns. The TSD + λ-MSD feature pair experiment had a percentage of bi-labeled trajectories of 38.3%, while the other experiments all had a percentage of bi-labeled trajectories of 26.7%.

**Table 3:** Spectral clustering results for the dataset of cell trajectory segments.

	Silhouette coefficient	Bi-labeled trajectories (%)
Feature collection	0.667	**26.7%**
TSD + λ-MSD	**0.673**	38.3%
TSD + mean speed	0.667	**26.7%**
λ-MSD + mean speed	0.667	**26.7%**

**Fig 6:**
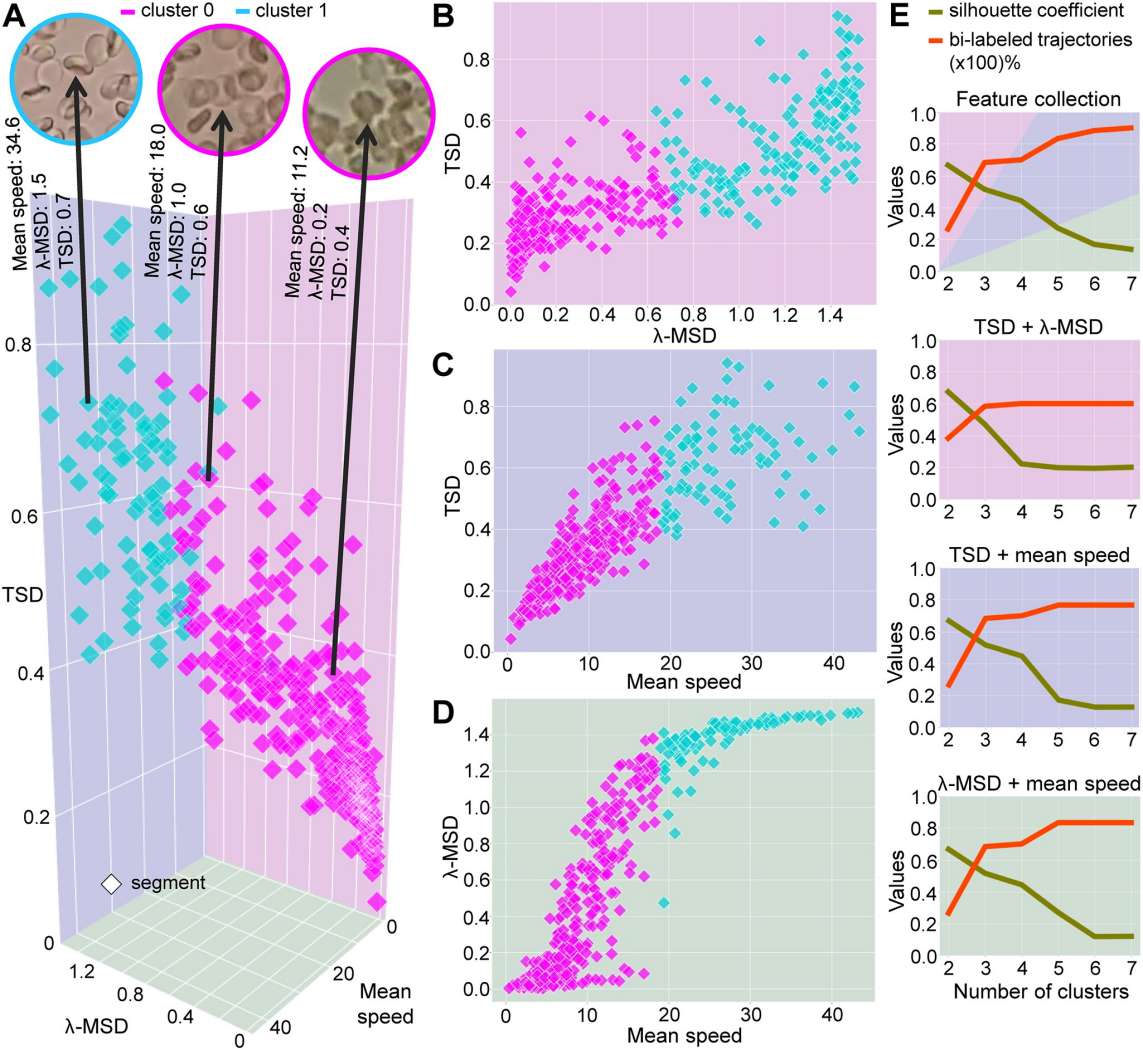
Clustering analysis of blood cell trajectory segments. Clusters obtained in the experiments: (A) the feature collection, in which the moving cell region is highlighted for some trajectory segments; (B) TSD + λ-MSD; (C) TSD + mean speed; and (D) λ-MSD + mean speed. (E) Behavior of silhouette coefficient and percentage of bi-labeled trajectories across different numbers of clusters for the studied feature combinations. Notably, the experiments reached the best results with two clusters, as we observed the highest silhouette coefficient value in conjunction with the lowest percentage of bi-labeled trajectories.

Our cluster analysis in Fig 6A–6D demonstrates that trajectory segments with low feature values tend to be labeled as cluster 0. Otherwise, the segments are labeled as cluster 1. We observe scattered segments in the frontier region, with different cluster assignments depending on the experiment. Note that the trajectory segments with a TSD value greater than 0.6 were assigned to cluster 0 in the three-dimensional analysis of the feature collection and to cluster 1 in the TSD + λ-MSD feature pair experiment. These segments contribute to intra-cluster cohesion in the TSD + λ-MSD feature pair experiment, increasing the silhouette coefficient. However, in this experiment, we observed that segments of the same trajectory are labeled differently, increasing the percentage of bi-labeled trajectories. In this case, we prefer a low percentage of bi-labeled trajectories since it suggests a certain tolerance of the motion pattern, even with small variations in the features. Fig 6E confirms that defining two clusters for the spectral clustering algorithm is optimal for identifying cell motions in optical microscopy videos, resulting in the highest value of the silhouette coefficient and the lowest percentage of bi-labeled trajectories. From this number of clusters, our experiments reached the best results regarding labeling cell trajectory segments, supporting the findings reported by Martins et al. [29].

In the feature collection experiment, the region where the cells move can reveal more details about the clustering results. Fig 6A shows three trajectory segments, with the cell region in the video frame highlighted. We chose trajectory segments close to the centroid of each cluster, in addition to a third segment in the frontier region between the clusters. In a quasi-confinement situation, the cell of the trajectory segment assigned to cluster 0 is in front of a cell clump. The cell labeled as cluster 0 in the frontier region has a moderate number of neighboring cells. It overlaps with some cells in the scene. The cell of the trajectory segment labeled as cluster 1 interacts with a smaller number of cells, and there is no overlap. Physical obstacles can make it difficult for cells to move or even prevent their motion. This dynamic context results in low values for the features, increasing the number of trajectory segments assigned to cluster 0.

**Fig 7:**
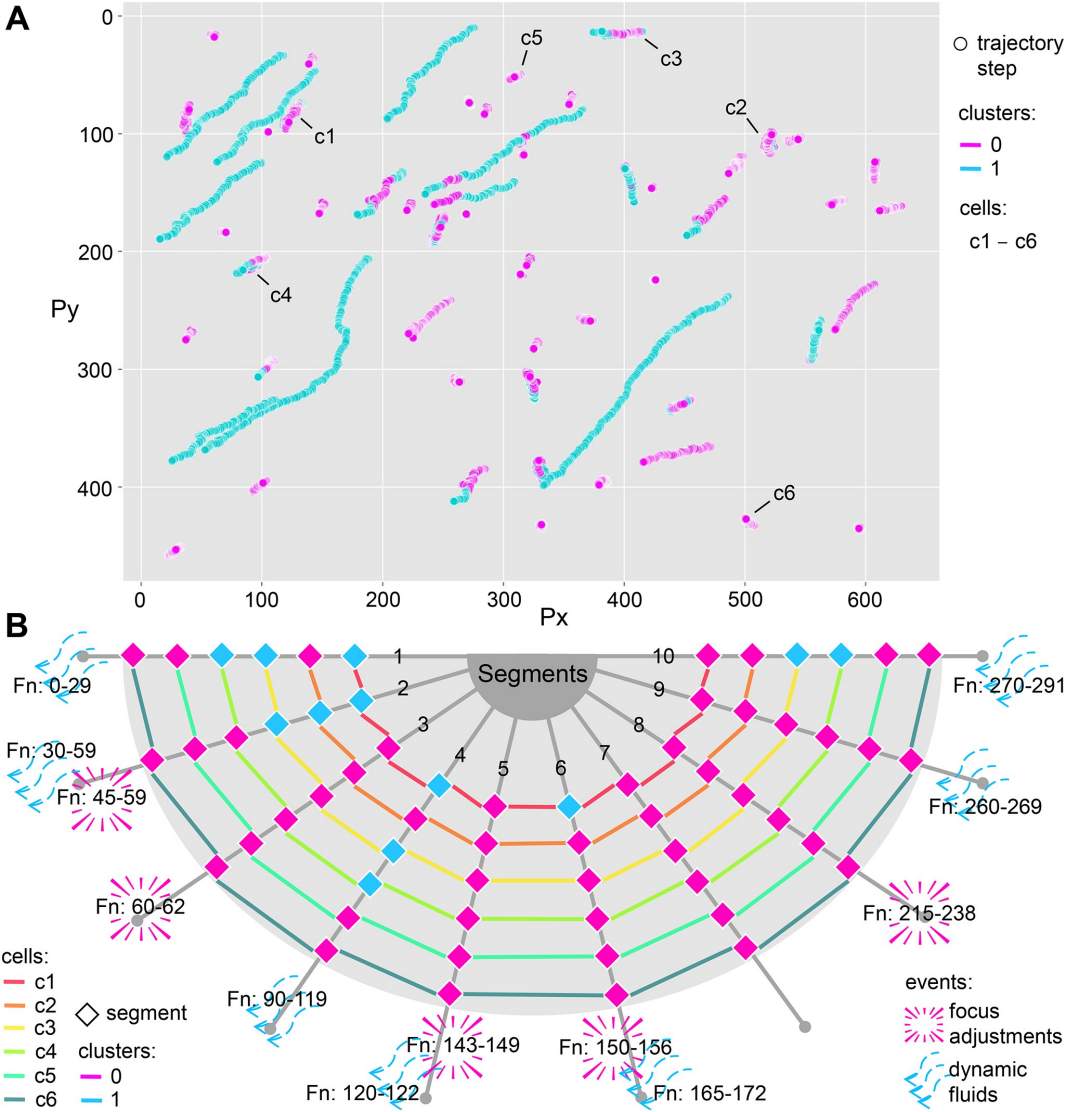
Analysis of bi-labeled trajectories in the feature collection experiment. (A) Complete cell trajectories with labeled steps according to segment clustering. The trajectories of cells c1 to c6 are analyzed below. (B) Influence of dynamic events on the occurrence of bi-labeled trajectories. The graph’s axes correspond to the trajectory segments, and the lines of specific colors represent the cells. The color of the rhombus indicates the clustering label. Dynamic events, which can focus on adjustments or dynamic fluids, are signaled at the end of each axis. We also highlight the frame number in which the event started and ended.

Fig 7A shows the set of cell trajectories, where each step is labeled according to the clustering results from the feature collection experiment. The cells are distributed in various regions of the microscope’s visual field, and two motion patterns are observed. Some cells move for small distances and tend to be assigned to cluster 0. Their trajectory is restricted and limited, a characteristic behavior of PTZ motion. The other cells move for greater distances and are assigned to cluster 1. Their trajectory is uniform and directional, following the displacement of blood fluids or solutions used in the *T. cruzi* parasitological analysis, as observed in the fluctuating motion. Such behaviors confirm the previous results, indicating that the clusters emphasize distinct motion patterns for cells. We also analyzed some bi-labeled trajectories from Fig 7A. We investigated whether cells c1 to c6, which are from the same video, change their motion pattern given a dynamic event. These events can have the participation of the elements or not. When the slide’s static equilibrium is affected, the elements tend to move under the action of fluids. Or even some stimuli, such as the microscope focus adjustments, introduce dynamicity without interacting with the elements.

The influence of dynamic events on the trajectories is shown in Fig 7B. The video containing cells c1 to c6 comprises 313 frames, resulting in 10 trajectory segments, each consisting of 30 steps. Based on our observation of the scene, we identified that the predominant motion of the cells is PTZ motion. This finding is further supported by clustering the trajectory segments of cells c1 to c6, which were mostly assigned to cluster 0. This indicates that dynamic events in the video stimulate the transition in the cells’ motion pattern toward fluctuating motion. An interesting aspect is that not all cells are influenced. Cells c5 and c6 did not change their labels over time. Furthermore, the motion pattern does not change for all elements simultaneously, e.g., only cells c3 and c4 change to cluster 1 due to dynamic fluid in the last trajectory segment. This evidences that some regions of the sample are more susceptible to fluid action. The stimulus duration and intensity must also be considered for changing the motion pattern. The dynamic fluid in segments 5 and 9 does not change the motion pattern of the cells, as this event is not present in many video frames. Cell c1 changes its label in segment 6, despite the short duration of the dynamic fluid. However, the event time is longer than that in segment 5 and is under the influence of focus adjustment compared to segment 9.

Our contribution to medical diagnosis involves applications specifically targeted at diagnosing acute Chagas disease, as the diagnosis in the chronic phase depends on serology. Other limitations of our work include the dynamism of the video, which, in very turbulent scenarios, can substantially change the trajectory of the elements. In addition, cells in contact with parasites can be influenced by them, assuming characteristics of the collateral motion. Although it contributes to detecting *T. cruzi* parasites, this behavior does not configure a regular cell motion pattern. Thus, we limited the work to confirm the initial hypothesis.

## Conclusions

This paper presents a trajectory-based analysis for the element characterization and identification of motion patterns in optical video microscopy. The elements in *T. cruzi* video microscopy present distinct dynamic patterns. Parasites perform the collateral motion, which is the most salient motion on the scene. Cells exhibit the PTZ motion, which leads to some displacement into a constrained area. Cells under fluctuating motion tend to follow a homogeneous and directional large path according to fluids in the blood sample. By measuring the standard deviation between trajectory steps of a moving element, the proposed TSD enables the discrimination between parasites and blood cells, outperforming state-of-the-art approaches. The collateral motion of the parasites has a superdiffusive behavior, and the interactions with the cells result in very random trajectory steps. When combined with mean speed and λ-MSD features, TSD enhances the identification of the cell motion pattern into fluctuating and PTZ, providing reasonable cohesion and separation between groups in our clustering experiments. Since fluctuating and PTZ motions tend to have low variance between the length of constant time trajectory steps, TSD performs poorly in differentiating these motions, with λ-MSD representing the most relevant feature in our surveyed feature collection. We confirm that λ-MSD captures explored area by the element, which is relevant to distinguishing a motion driven by a flow from that occurring in a constraint region. The dynamic events, such as fluids and microscope focus adjustment, may lead to bi-labeled cell trajectories. The effects of these events on cells depend on the microscope’s field of view, the cell positioning in the blood slide, and the stimulus’s intensity and duration. Since that Chagas disease is a neglected disease, our findings may contribute to developing new computational tools focused on motion, boosting the study and medical diagnosis of Chagas disease. Future works may investigate the interaction mechanisms between parasites and cells, providing insights into if and how cells may assume collateral motion. The trajectory patterns of elements also may be analyzed under the action of drugs or other substances.
